# Chitosan Membranes for Direct Methanol Fuel Cell Applications

**DOI:** 10.3390/membranes13100838

**Published:** 2023-10-20

**Authors:** Livhuwani Modau, Rudzani Sigwadi, Touhami Mokrani, Fulufhelo Nemavhola

**Affiliations:** 1 Department of Chemical Engineering, University of South Africa, Florida 1710, South Africa; 12739634@mylife.unisa.ac.za (L.M.); sigwara@unisa.ac.za (R.S.); tmokrani@unisa.ac.za (T.M.); 2 Department of Mechanical Engineering, Faculty of Engineering and the Built Environment, Durban University of Technology, Durban 4000, South Africa

**Keywords:** chitosan, fuel cell, water uptake, proton conductivity, silica/chitosan, composite membranes, methanol permeability, solution casting, direct methanol fuel cell

## Abstract

The purpose of this study is to identify the steps involved in fabricating silica/chitosan composite membranes and their suitability for fuel cell applications. It also intends to identify the physical characteristics of chitosan composite membranes, including their degree of water absorption, proton conductivity, methanol permeability, and functional groups. In this investigation, composite membranes were fabricated using the solution casting method with a chitosan content of 5 g and silica dosage variations of 2% and 4% while stirring at a constant speed for 2 h. According to the findings, the analysis of composite membranes produced chitosan membranes that were successfully modified with silica. The optimum membrane was found to be 4% s-SiO_2_ from the Sol-gel method with the composite membrane’s optimal condition of 0.234 cm/s proton conductivity, water uptake of 56.21%, and reduced methanol permeability of 0.99 × 10^−7^ cm^2^/s in the first 30 min and 3.31 × 10^−7^ in the last 150 min. Maintaining lower water uptake capacity at higher silica content is still a challenge that needs to be addressed. In conclusion, the fabricated membranes showed exceptional results in terms of proton conductivity and methanol permeability.

## 1. Introduction

The increasing scarcity of fossil fuels has highlighted the need for alternative energy sources [1,2,3]. Fossil fuels are not renewable, which makes them a threat to energy security. The use of these non-renewable resources also endangers the lives of individuals as they release harmful greenhouse gases like methane and carbon monoxide into the atmosphere [4]. Reducing our dependency on fossil fuels by switching to alternative energy sources like fuel cells increases energy stability and security [5]. Fuel cells can work well with other clean energy sources, such as methanol. Depending on the electrolyte’s type, the fuel cell can be categorized into different types, such as proton exchange membrane fuel cells, also known as PEMFCs, solid oxide fuel cells (SOFCs), alkali fuel cells (AFCs), and molten carbonate fuel cells (MCFCs) fuel cells [4,6,7,8]. The PEMFC is one of the most widely used energy techniques in the world due to its high energy conversion efficiency and lack of pollution [9,10]. Direct methanol fuel cell (DMFC) is a promising technology that is expected to revolutionize the way we produce and use electricity [4,9]. DMFCs have attracted widespread attention due to their unique attributes, such as their low emissions, easy liquid fuel storage, and high energy density [11,12]. DMFCs are based on liquid-fuel technology and utilize direct methanol as their fuel for electricity generation [13]. They are market leaders in the field and are commonly utilized in mobile and off-grid power applications [14,15]. Polymer membranes are the main components of direct methanol fuel cells [16,17,18]. DMFCs must be designed to provide high ion exchange capacity and low water uptake. They should also have good proton conductivity and a long life span [19,20]. The high proton conductivity of PEM ensures that it can conduct protons efficiently from the anode to the cathode. Its robust fuel barrier helps prevent degradation or even the termination of fuel cell performance [8]. Its good mechanical properties ensure that it operates in both wet and dry environments. Today’s membranes are made from perflurosulphonic acids, which are commonly referred to as Nafion. Unfortunately, Nafion has drawbacks such as high methanol permeability and high cost, which also contribute to its application in fuel cells [20,21]. Natural and low-cost, abundant chitosan-based polymers can be utilized as an alternative in fuel cells. However, due to their high hydrophilic nature and low proton conductivity, they are not ideal for DMFC applications [4]. The biopolymer chitosan is made from chitin, which is found in the shells of insects and crustaceans. With the help of hygroscopic oxide fillers such as silica (SiO_2_), titanium dioxide (TiO_2_), and zirconium oxide (ZrO_2_), they can achieve good membrane properties such as high proton conductivity, low water uptake, high membrane selectivity, and reduced methanol permeability [22,23]. Several studies have also shown that chitosan membranes made with pure/sulfonated silica exhibit better proton conductivity and water uptake than those made without this modification. Chitosan membranes are commonly modified with silica nanoparticles to enhance their mechanical strength, stability, and barrier properties. The addition of silica can improve the overall performance of the membrane by reducing the permeability of certain molecules, including methanol [22,23]. The chitosan structure has two major groups: the amino and the hydroxy groups, which makes it easy to modify. Depending on the modification process, chitosan can undergo physical and chemical transformations [24]. Chemical modification can be performed on either the amino or the hydroxy groups, depending on the reaction [24]. Although the primary group of chitosan reacts actively to the amino group, it is less reactive than the secondary group. When a chemical modification is carried out on the amino group, it will be labeled as N, while it will be O modified on the other side. Physical methods can be utilized to modify chitosan, such as mechanical grinding, ultrasonic treatment, and ionizing radiation [25]. DMFC utilizes different types of membranes based on their morphological attributes. These include thick, thin, layered, porous, and pore-filled membranes [26]. Although the properties of chitosan can be modified to improve its suitability for fuel cells, membrane morphology can still have a significant impact on its performance. For example, DMFC’s thin membranes are prone to experiencing high methanol crossover when compared to thick ones. In this manuscript, modification of the chitosan membrane with silica was found to improve the membrane proton conductivity and ion exchange while reducing methanol permeability.

## 2. Experiment

### 2.1. Materials

Chitosan flakes, Medium molecular weight, (Merck, Rahway, NJ, USA), Tetraethyl orthosilicate, Si(OC_2_H_5_)_4_, 98%, (Merck), Ammonia, NH_3,_ 25%, (Merck), Acetic acid, CH_3_COOH, 99%, (Merck), Sodium hydroxide, NaOH, (Merck), Ethanol, C_2_H_5_OH, 99.9%, (Merck), Methanol, CH_3_OH, 99.9% (Merck), Sodium chloride, NaCl, (Merck), Hydrochloric acid, HCl, 37%, (Merck), Sulfuric acid, H_2_SO_4_, 99%, (Merck), Taurine, C_2_H_7_NO_3_S, 99%, (Merck).

### 2.2. Synthesises of SiO_2_ Nanoparticles

(i) Sol-gel Process

The process for producing silica particles using tetraethyl orthosilicate involves the condensation and hydrolysis of TEOS. Various substances, such as 200 mL of ethanol, 80 mL of TEOS, and ammonia, were stirred at varying temperatures for around 30 min. A substance resembling a sol-gel was then dried at a rate of 100 °C for a single day. The silica particles were then catalyzed to remove impurities by calcinating them at a rate of 600 °C for 2 h. The dried silica particles were stored in sample bottles for use in membrane fabrication.

(ii) Stober Process

The precursor for silica was TEOS during the Stober process, which involved the addition of ammonia, water, and ethanol. The mixture, which was then centrifuged for over 30 min, was then purified using a gasifier. The white solid materials, which were dried at a rate of 100 °C for 24 h, were then subjected to a similar process as those of Sol-gel.

### 2.3. Sulfonation Process

Sulfuric acid was used to prepare silica nanoparticles. These particles were sulfonated to improve their proton conductivity. The process used for producing sulfonated silica nanoparticles for the Sol-gel and Stober processes was the same. In a mixing process, 10 g of silica particles synthesized in Section 2.2 (i and ii) were mixed with 5 mL of sulfuric acid and 200 mL of methanol. At 1500 rpm, the mixture was vigorously stirred. It was then subjected to a continuous centrifuge for about 31 min. The centrifuged silica particles were separated from the liquid solution and subjected to a drying process before they were stored.

### 2.4. Fabrication of Composite Membranes (Casting Method)

Chitosan flakes were used to make chitosan membranes. The casting technique was used to create the membranes. Chitosan flakes weighing 5 g were added to a 2% *v*/*v* acetic acid solution and agitated for an hour. Dimethyl sulfoxide (DMSO), taurine/2-aminoethanesulfonic acid (C_2_H_7_NO_3_S), and silica particles (SiO_2_/s-SiO_2_) at different weight ratios (2% or 4%) were also added. 250 mL of the chitosan gel solution was cast into porcelain plates and then dried at 70 °C for 5 h. The acquired dried membrane was cross-linked with 2 M sulfuric acid for 30 min before being submerged in NaOH. The membrane surface was then cleansed with deionized water to remove any surplus acid, then dried. The dried membranes produced have a membrane thickness of 0.041 cm. silica-chitosan membranes were also processed in this manner.

## 3. Characterization Techniques

### 3.1. Water Uptake 

The water uptake ratio is a measure of how much water can be absorbed by a membrane. This is very important in the design and construction of fuel cell membranes. The water uptake rate is also a factor that contributes to the membrane’s swelling capacity. Cell efficiency can be affected by high water uptake. The difference in the membranes’ mass between those that are dehydrated and hydrated is used to determine how much water they take up. Two independent experiments were used to calculate the water uptake of chitosan membranes having dimensions of 2 cm × 2 cm at room temperature. The mass of the membrane in a dry state (W_dry_) was measured before it was soaked in water for 24 h. The mass of the wet state membrane (W_wet_) was obtained after the membrane was soaked in distilled water for 24 h, then wiped with a paper towel to remove moisture. Water uptake was calculated as follows:

(1) $$\mathrm{Water}\;(\%)=(\frac{\mathrm{Mw}-\mathrm{MD}}{\mathrm{MD}})\times 100\%$$

where M_W_ is described as the weight of wet membranes and M_D_ is the dry membrane’s weight.

### 3.2. Ion Exchange Capacity (IEC)

The ion exchange capacity was measured using the method used by [27]. The dried membranes were soaked in 20 mL of a 2 M NaCl solution for 24 h for Na^+^ to interchange H^+^ ions. The H^+^ ion solution was titrated with a solution of sodium hydroxide using a phenolphthalein indicator. The protons that were released were titrated with 0.01 M NaOH until they reached the end point. Ion exchange capacity was calculated as follows:

(2) $$\mathrm{IEC}=\frac{\mathrm{VNaOH}*\mathrm{CNaOH}}{\mathrm{Mdry}}$$

where IEC is the ion exchange capacity of the membrane, C_NaOH_ is- the concentration of NaOH in mol/L, V_NaOH_ is- the volume of NaOH that will be used to neutralize H+ in mL, and Mdry is- the mass of the dry membrane in g.

### 3.3. Methanol Permeability

Membrane permeability to methanol was determined at room temperature by a two-compartment diffusion cell with the membrane in between. Both A and B cell compartments were loaded with the same amount of methanol fuel and water, respectively. Both sides of the cell were stirred. Permeability values were calculated as follows:

(3) $$P=\frac{L}{A}\times \frac{V_{b}}{C_{a}}\times \frac{\Delta C}{\Delta t}$$

P is the permeability thickness of the membrane, A is the area available for diffusion in the membrane volume of receiving compartment, Ca is the concentration of sample in component A, ΔC is the change in methanol concentration, and Δt is the permeation time.

### 3.4. Brunauer-Emmett-Teller (BET) Measurements 

The BET analysis was carried out using the micrometric 3-Flex system. After the gas has been purged, a dry sample will be cooled down to around 77 K using liquid nitrogen. The inert gas will then adhere to the surface of the sample and lower the pressure inside the analysis chamber [28,29]. Then the adsorption isotherm of the experiment was used to determine the surface area of the samples. All samples were degassed under a pressure of around 3 × 10^−5^ mbar. Particle size can be determined using the following equation:

(4) $$S=\frac{6}{\rho D_{BET}}\times 10^{3}$$

ρ—denote the theoretical density of the material, and $D_{BET}$—denote the size of the particle in nm.

### 3.5. X-ray Powder Diffraction Analysis (XRD)

XRD includes structures, preferred crystal orientations (texture), and additional structural elements such as crystallinity, average grain size, crystal flaws, and strain. The constructive interference of a monochromatic beam of X-rays dispersed at precise angles from each pair of a sample’s lattice planes results in the formation of X-ray diffraction peaks. The arrangement of atoms within the lattice affects peak intensities. As a result, each material’s periodic atomic configurations are identified by its X-ray diffraction peaks. According to Bragg’s law, constructive interference (and a diffracted ray) take place when incident rays come into contact with a sample [30]. Bragg’s law equation:

(5) nλ=2dsinθ

This theory relates the diffraction angle and lattice spacing of a crystalline sample to the electromagnetic radiation spectrum. After that, the diffracted X-rays are detected, examined, and counted. Due to the non-uniform dispersion of the powdered material, scanning the sample at a range of 2 should reveal all possible lattice diffraction orientations. The chemical can be recognized by converting the diffraction peaks to d-spacings, even though each material has a unique set of d-spacings. This is typically accomplished by comparing d-spacings to recognized reference patterns.

## 4. Results and Discussion

### 4.1. Fourier Transform Infrared for Chitosan Membranes

**Figure 1:**
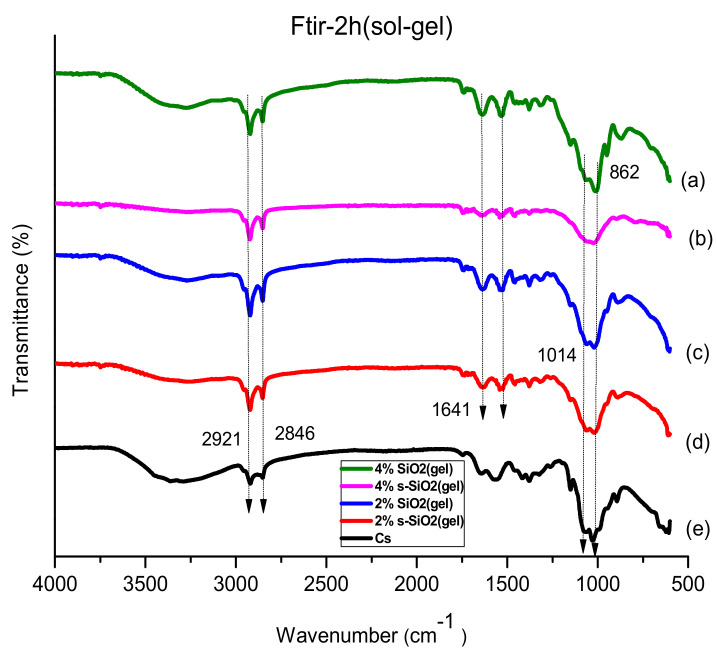
FTIR of (**a**) 4% s-SiO_2_/Cs, (**b**) 4% SiO_2_/Cs, (**c**) 2% SiO_2_/Cs, (**d**) 2% s-SiO_2_/Cs, and (**e**) Cs–Sol-gel (2 h).

**Figure 2:**
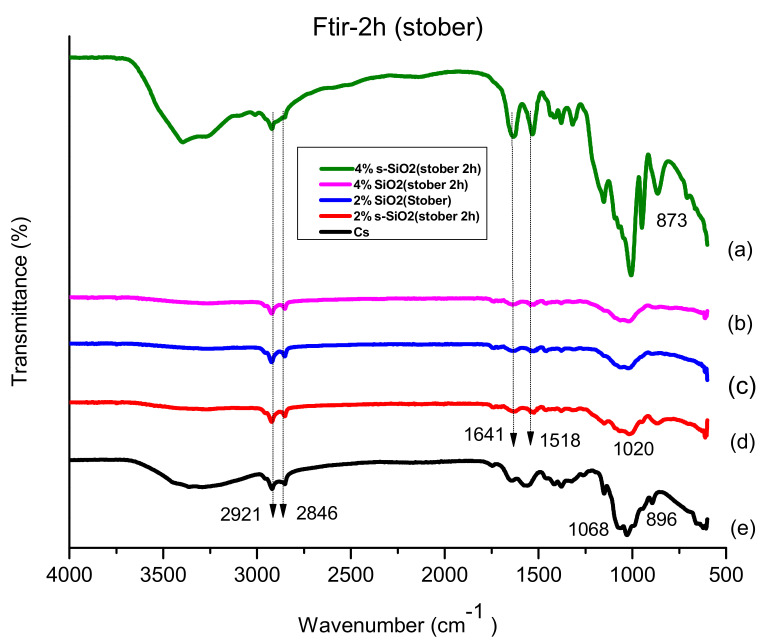
FTIR of (**a**) 4% s-SiO_2_/Cs, (**b**) 4% SiO_2_/Cs, (**c**) 2% SiO_2_/Cs, (**d**) 2% s-SiO_2_/Cs, and (**e**) Cs–(Stober 2 h).

FTIR spectra were used to analyze the SiO_2_ creation in the hybrid chitosan-silica composite. The spectra were obtained for both the modified and pure chitosan membranes. The analysis of the membranes using FTIR is shown in Figure 1 and Figure 2, which revealed that the membranes exhibited a peak between 3370 and 3250 cm^−1^. This peak represents the presence of the N-H group, which has an overlapping relationship with the O-H group [31]. The peak was shifted in the composition of modified chitosan membranes from 3331 cm^−1^ to (a and b) 3338 cm^−1^, (c) 3294 cm^−1^, and (d) 3333 cm^−1^ of Figure 1, and in Figure 2 it shifted to (a) 3343 cm^−1^, (b) 3379 cm^−1^, (c) 3409 cm^−1^, and (d) 3428 cm^−1^. The shift in the peak shows that the interaction between the N-H of chitosan and silica’s O-H groups is occurring. The peaks at 2846 cm^−1^ and 2921 cm^−1^ are respectively related to the vibrations produced by the C-N and C-H stretching [32]. The CS and the CS/(SiO_2_/s-SiO_2_) membrane’s amino group’s N-H bond deformation is at 1518 cm^−1^. The absorption bands found on the Stober (1020 cm^−1^) and Sol-gel (1014 cm^−1^) membranes are believed to be caused by Si-O-S vibration. On the other hand, the absorption bands found on the 873 and 862 cm^−1^ membranes were attributed to Si-OH [33] Although the spectra did not show a new functional group, the membranes made by sol-gel exhibited high-intensity peaks, which is a result of their interaction with chitosan and silica.

### 4.2. X-ray Diffraction of Chitosan Membranes

**Figure 3:**
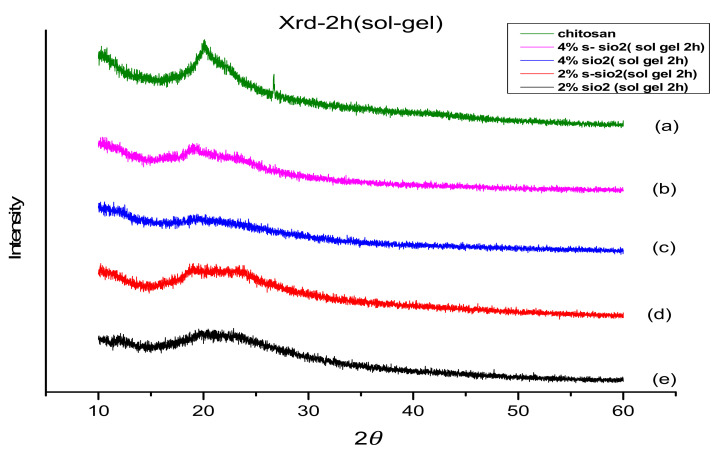
XRD of (**a**) Cs, (**b**) 4% s-SiO_2_/Cs, (**c**) 4% SiO_2_/Cs, (**d**) 2% s-SiO_2_/Cs, and (**e**) 2% SiO_2_/Cs–Sol gel (2 h).

**Figure 4:**
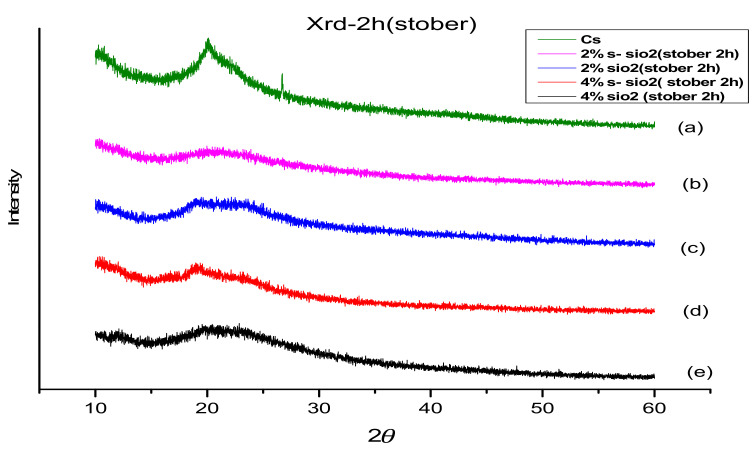
XRD of (**a**) 2% SiO_2_/Cs, (**b**) 2% s-SiO_2_/Cs, (**c**) 4% SiO_2_/Cs, (**d**) 4% s-SiO_2_/Cs, and (**e**) Cs–(Stober 2 h).

The interactions between the chitosan molecules’ intramolecular and intermolecular components contribute to their crystallinity. The X-ray diffraction (XRD) method was used to determine SiO_2_’s impact on the crystallinity of CS. Figure 3 and Figure 4 show the XRD analysis of various types of membranes, including 2% s-SiO_2_ and 4% s-SiO_2_. The chitosan membrane exhibits semi-crystalline characteristics at 19°. The modified membranes exhibited an amorphous structure with different angles of refraction, such as 19.78, 20.29, 21.26, and 21.35 degrees, as shown in Figure 3 and Figure 4. Figure 3 and Figure 4 show the crystallinity loss experienced during the modification process. The figure shows the decline in the crystallinity peak of the modified chitosan membranes. The addition of silica fragments containing sulfuric acid caused the modified membranes to have an amorphous structure and decreased the total crystallinity.

### 4.3. SEM of Chitosan Membranes

**Figure 5:**
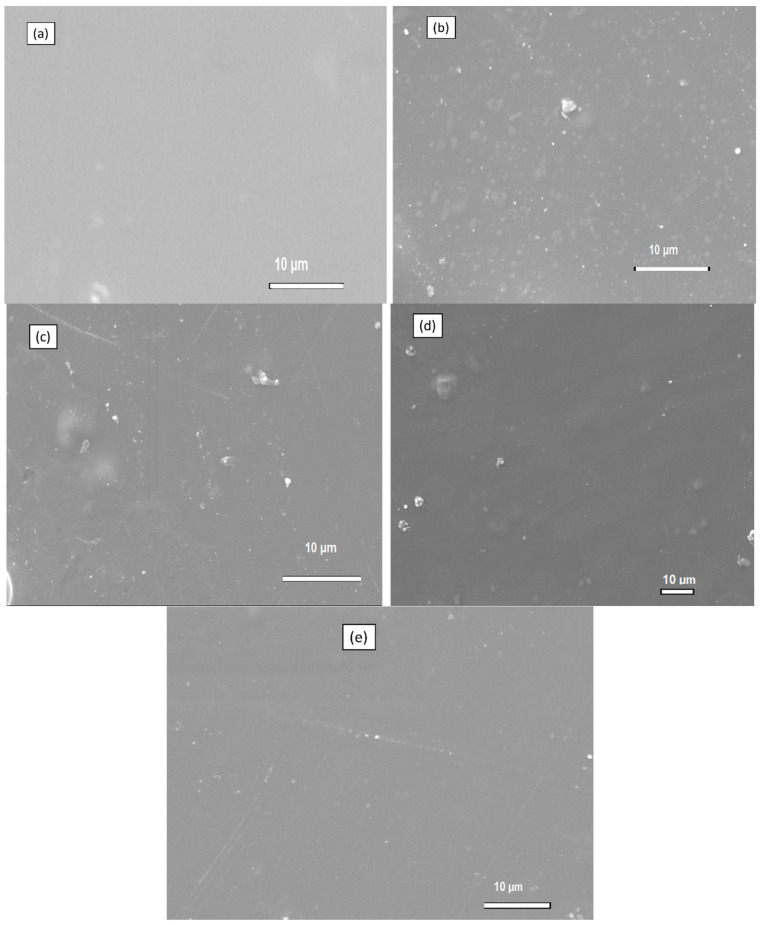
SEM of (**a**) Cs, (**b**) 4% s-SiO_2_/Cs, (**c**) 4% SiO_2_/Cs, (**d**) 2% s-SiO_2_/Cs, and (**e**) 2% SiO_2_/Cs–Sol-gel (2 h).

**Figure 6:**
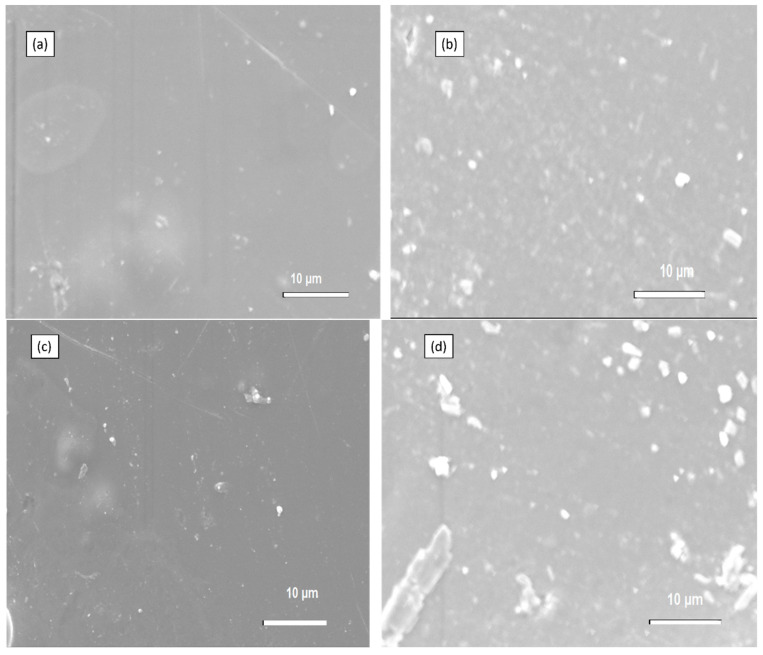
SEM of (**a**) 2% s-SiO_2_/Cs, (**b**) 2% SiO_2_/Cs, (**c**) 4% s-SiO_2_/Cs, and (**d**) 4% SiO_2_/Cs-Stober (2h).

Figure 5 and Figure 6 indicates the morphologies of the chitosan membranes. The changes in Chitosan’s morphology were observed using SEM. These transformations were observed before and after SiO_2_ modification. The image in Figure 5a shows a smooth and complex surface that does not have any visible pores on it. The results support the findings of a study conducted by Riek and colleagues in 2017, who discovered that the chitosan membrane has uniform and homogenous flat surfaces and a consistent thickness. A lot of silica nanoparticles are visible in membranes a and b, while in membranes c and d, a little amount of silica was detected in Figure 5. This is due to the number of silica nanoparticles that were added to the membranes, as membranes a and b have a high silica content compared to those of c and d. It is evident that although silica nanoparticles were dispersed, they were not evenly distributed in the polymer matrix of the membrane. It also appears that the dispersed nanoparticles form aggregates, and the more silica added, the higher the number of agglomerated silica nanoparticles. The high energy level of silica particles causes silica agglomeration. However, modification of silica with sulfur seems to reduce this agglomeration since membranes b and e have lower agglomerates compared with those of their unmodified silica membranes.

The physical morphologies of membranes with silica synthesized through Stober are indicated in Figure 6 The SEM images indicate membranes with dense morphologies and hard surfaces. Membranes with silica from Stober have similar morphologies to those of Sol-gel. However, membrane (a) (2% SiO_2_) in Figure 6 has lumps on it, which may have developed during the drying process, but these lumps are not visible in other membranes. When looking at membrane c in Figure 6, it can be observed that it has a better distribution of silica on its surface, but agglomeration is also visible. The agglomeration phenomena have occurred in all of the fabricated membranes (Sol-gel and Stober) and membranes with silica synthesized, though Stober has more agglomerates on its surface compared to those of Sol-gel. The challenge is still to synthesize silica particles that will form little to no agglomerates for the modification of chitosan.

### 4.4. Water Uptake of Chitosan Membranes

(i) Effect of Silica Content on Water Uptake

**Figure 7:**
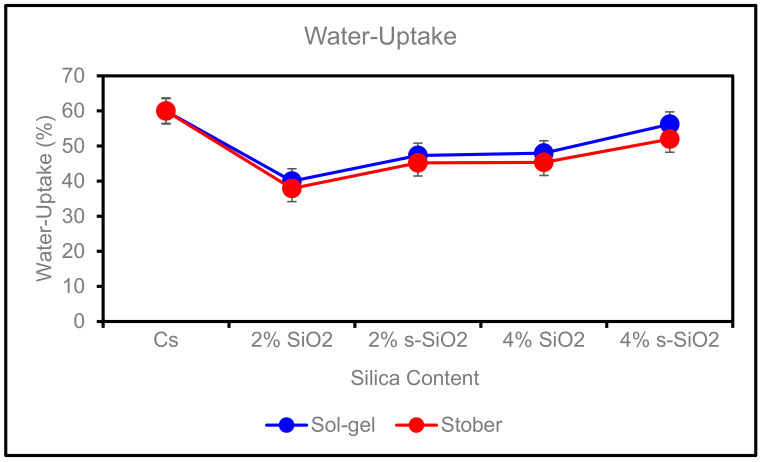
Effect of silica content on water uptake of Cs, 2% s-SiO_2_/Cs, 2% SiO_2_/Cs, 4% s-SiO_2_/Cs, and 4% SiO_2_/Cs–Sol-gel (2 h) and Stober (2 h).

The membrane’s water uptake is a vital part of its physical properties. The water uptake of chitosan membranes as a function of silica (SiO_2_/s-SiO_2_) is shown in Figure 7. The chitosan contains different silica contents of 2% and 4%. It can be seen that the incorporation of silica into the chitosan matrix suppresses the water absorption of the membrane uptake by 22% on 2% SiO_2_/Cs; however, adding more silica content to the membrane resulted in an increase in water uptake as membranes containing 4% SiO_2_ had higher water uptake of 45.34% and 47.98%, whereas those of the corresponding 4% s-SiO_2_ had 51.97% and 56.21% Stober and Sol-gel, respectively. The increase in water uptake at higher silica levels can be attributed to silica hygroscopic affinity and bonding interaction between the silanol group and chitosan amine, acetyl, and silanol groups interacting more strongly and increasing membrane hydrophilicity [34]. The increase in water uptake of chitosan membranes containing high silica content can be attributed to the high water affinity of silica particles, which contribute to the total hydrophilic property of the membrane. As chitosan also has a high hydroscopic property, this caused the membrane’s overall water uptake to be high [35]. The highest water uptake is 56.21%, belonging to the 4% s-SiO_2_ membrane. Higher values in s-SiO_2_ membranes are due to the presence of sulfonic acid groups on the surface of the silica particles, which can interact with water molecules and promote their uptake. These findings indicate that making use of chitosan modified with a small quantity of silica can lead to significant improvements in the membranes’ water uptake capacities, potentially affecting a wide range of applications.

(ii) Effect of Temperature on Water Uptake

**Figure 8:**
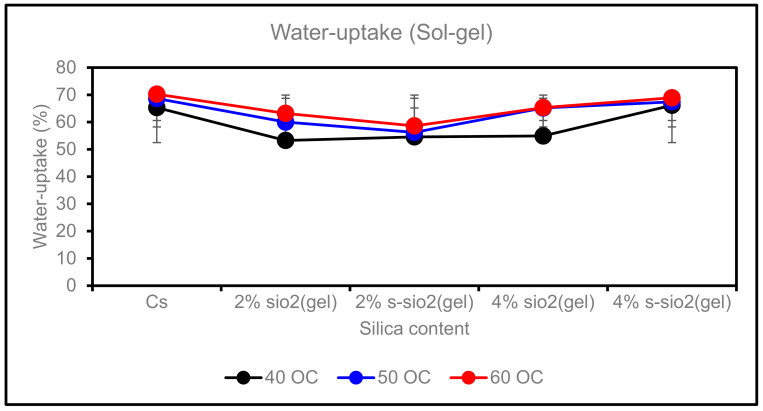
Effect of temperature on water uptake of Cs, 2% s-SiO_2_/Cs, 2% SiO_2_/Cs, 4% s-SiO_2_/Cs, and 4% SiO_2_/Cs–Sol-gel (2 h).

**Figure 9:**
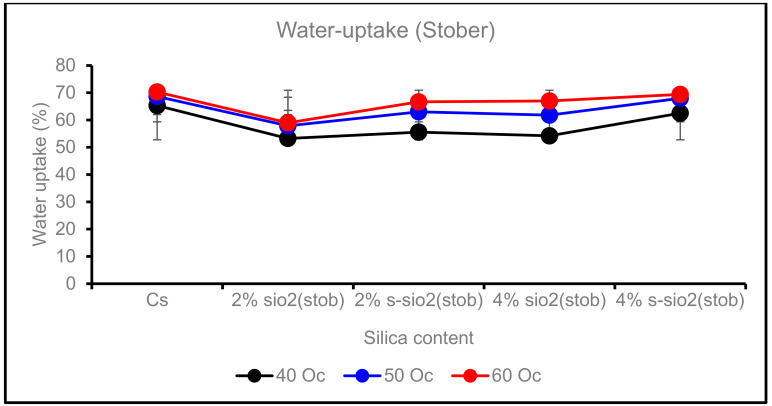
Effect of temperature on water uptake of Cs, 2% s-SiO_2_/Cs, 2% SiO_2_/Cs, 4% s-SiO_2_/Cs, and 4% SiO_2_/Cs–Stober (2 h).

Figure 8 represents the effect of temperature on the water uptake of chitosan membranes. The temperature can influence how the chitosan and silica composites will expand. This is because higher temperatures can cause the molecules of the solvent to expand into the matrix. The processing conditions and composition of the composite will determine how much swelling will occur. As shown above, in Figure 8, the membranes show an increase in water uptake when the temperature rises from 40 °C to 60 °C; however, the water uptake on modified membranes was lower than that of pure chitosan, recorded to be 60%. In Figure 9, modified membranes have uptake between 53.26 °C and 68.92 °C (Sol-gel), and those of Stober are 53.25 °C–69.39 °C at 40 °C–60 °C. The increase is due to membrane water diffusivity, chain mobility, and membrane-free volume. However, the 4% s-SiO_2_ Sol-gel membrane does not show a significant change from 40 to 60 °C, as its water uptake is 65.17% and 65.35% at 40 °C and 60 °C, respectively. The minimal water uptake rate on the 4% s-SiO_2_ membrane can be attributed to the chitosan and silica interaction as well as the lack of free void volume, which enables the membranes to endure high temperatures. It can be concluded that membranes with silica synthesized through Sol-gel have high water uptake compared to those of Stober, with s-SiO_2_/Cs membranes having the highest water uptake. This can reduce membrane application if the membranes become spongy, reducing their life span. In conclusion, Stober membranes’ low water uptake is an ideal property for fuel cell applications. Also, the minimal water uptake can improve their durability. 

### 4.5. Ion Exchange Capacity of Chitosan Membranes

**Figure 10:**
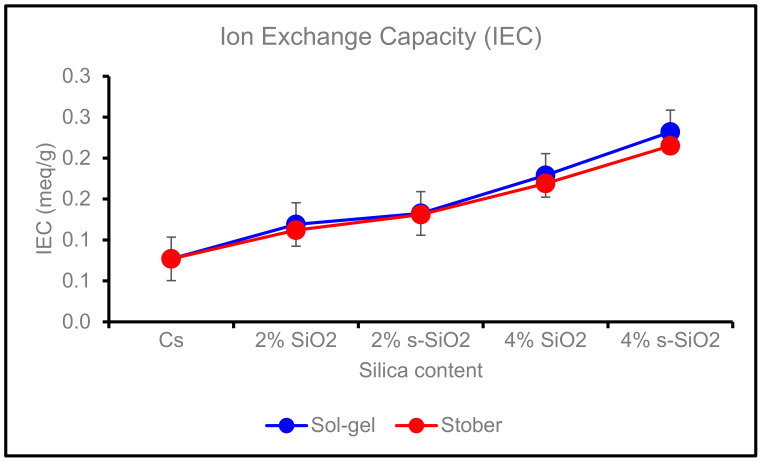
Ion exchange capacity of Cs, 2% s-SiO_2_/Cs, 2% SiO_2_/Cs, 4% s-SiO_2_/Cs, 4% SiO_2_/Cs, and Sol-gel (2 h) and Stober (2 h).

The ion exchange capacity of the membranes is illustrated in Figure 10. It is indicated in Figure 10 that pure chitosan has the lowest IEC of 0.77 meq/g, and the addition of silica improves the chitosan membrane’s IEC from 1.12 meq/g (2% SiO_2_) to the highest exchange value of 2.32 meq/g (4% s-SiO_2_). Sulfonated Sol-gel membranes have the highest IEC, and it is mainly due to the presence of acid groups caused by SiO_2_-SO_3_H particles’ acidity. The overall results indicate that incorporating silica nanoparticles can boast superior ion exchange capacities compared to their chitosan counterpart. The increase in IEC can be attributed to (i) chitosan having amino groups that can be deprotonated or protonated to allow for the exchange of ions, and (ii) the addition of functional groups in the chitosan layer that improves the site available for exchange [34]. (iii) The incorporation of silica nanoparticles into chitosan membranes can improve the surface accessibility of certain functional groups, and the silica’s structure facilitates the movement of ions into the matrix, enhancing the exchange of ions. (iv) The chemical and mechanical properties of chitosan-based membranes can be improved through the incorporation of silica, which will extend their service life and improve their ability to exchange ions. 

### 4.6. Proton Conductivity of Chitosan Membranes

**Figure 11:**
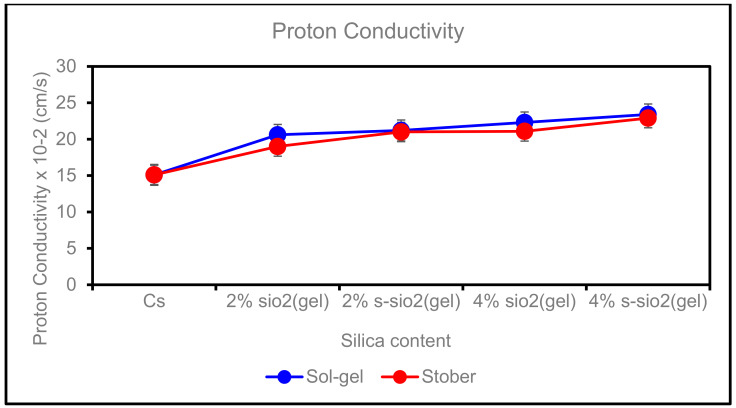
Proton conductivity of Cs, 2% s-SiO_2_/Cs, 2% SiO_2_/Cs, 4% s-SiO_2_/Cs, and 4% SiO_2_/Cs–Sol-gel (2 h) and Stober (2 h).

Figure 11 indicates the proton conductivity of the chitosan membrane at room temperature. The proton conductivity of the membrane is an important contributing factor in the application of membranes in fuel cells. It helps in determining if the membrane can produce the energy needed. The vehicle and the Grotthuss mechanisms are responsible for proton transfers in PEM [36]. In Figure 11, an increase in proton conductivity was observed when more filler was added. This is due to the influence of the ionic group Si-OH, which facilitates proton conduction. Chitosan was reported to have a proton conductivity of 0.151 cm/s and that of modified membranes (2% SiO_2_) having the lowest proton conductivity of 0.206 cm/s and 0.21 cm/s with the highest conductivity (4% s-SiO_2_) of 0.229 cm/s and 0.234 cm/s, Stober and Sol-gel, respectively. The high increase in proton conductivity of s-SiO_2_ Sol-gel membranes can be attributed to the high water uptake of the membranes reported in Figure 7, Figure 8 and Figure 9. The proton conductivity of PEM is affected by the presence of water as well as the dissociation of the groups of mobile protons and sulfonic groups; therefore, enhanced water uptake helps facilitate proton transfers through membranes’ ionic channels [37]. The reason why the conductivity of the membranes is outstanding is due to the presence of high-proton-conducting sulfonating compounds that are stuck in the routes of the sulfonating polymers and SiO_2_.

### 4.7. Methanol Permeability of Chitosan Membranes

**Figure 12:**
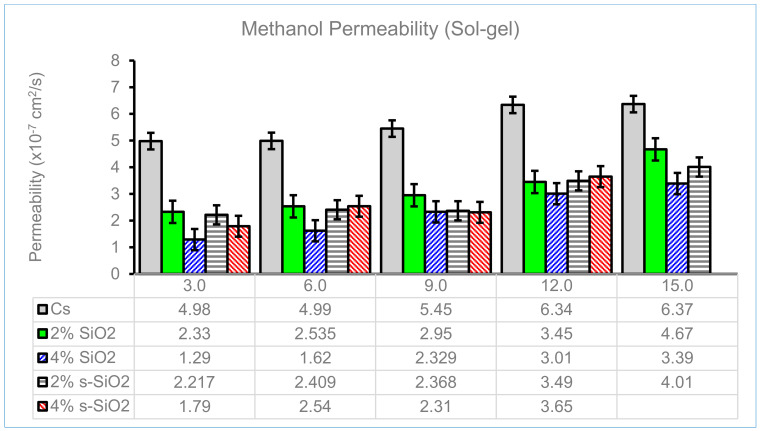
Methanol permeability of Cs, 2% s-SiO_2_/Cs, 2% SiO_2_/Cs, 4% s-SiO_2_/Cs, and 4% SiO_2_/Cs–Sol-gel (2 h).

**Figure 13:**
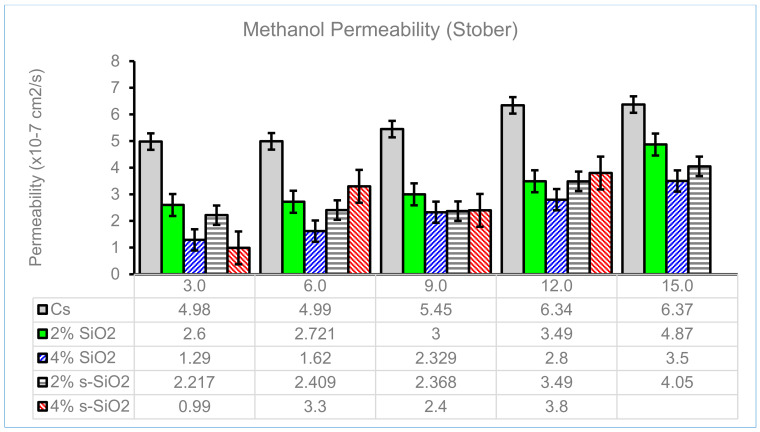
Methanol permeability of Cs, 2% s-SiO_2_/Cs, 2% SiO_2_/Cs, 4% s-SiO_2_/Cs, and 4% SiO_2_/Cs–Stober (2 h).

DMFCs are powered by methanol as fuel, and the low permeability of PEM will contribute to their efficiency. Figure 12 and Figure 13 display the influence of the silica particle content on the methanol permeability of the chitosan composite membranes, as well as the relationship between time and the permeability behaviors of a membrane at 30, 60, 90, 120, and 150 min. It shows that the permeability of the chitosan membrane increases as the time interval is increased from 30 to 150 min. Additionally, Figure 12 and Figure 13 show that silica nanoparticles in varying wt% reduce the crossover between the membrane and methanol. Figure 12 illustrates the lowest methanol permeability on the modified membrane, which is found on a 4% s-SiO_2_ membrane, with values of 1.79 × 10^−7^ cm^2^/s at 30 min and 2.31 × 10^−7^ cm^2^/s at 90 min. The decrease in permeability is due to ion-exchangeable acid groups that promote proton conduction by forming a hydrogen bond network that is strong enough to resist methanol permeability [38,39]. The permeability of the modified chitosan membrane decreases by almost 2% when compared to the unmodified chitosan membrane. This phenomenon is due to the cross-linked structure of the modified membranes. The Stober membranes have the lowest methanol permeability of 2.6 × 10^−7^ on a 2% s-SiO_2_ membrane for the first 30 min, as shown in Figure 13. Figure 13 demonstrates that as time increases, the methanol permeability on Stober membranes also increases to 0.99 × 10^−7^ at 30 min and 2.4 × 10 ^−7^ at 90 min on 4% s-SiO_2_ membranes, respectively.

### 4.8. Tensile Strength of Chitosan Membranes

**Figure 14:**
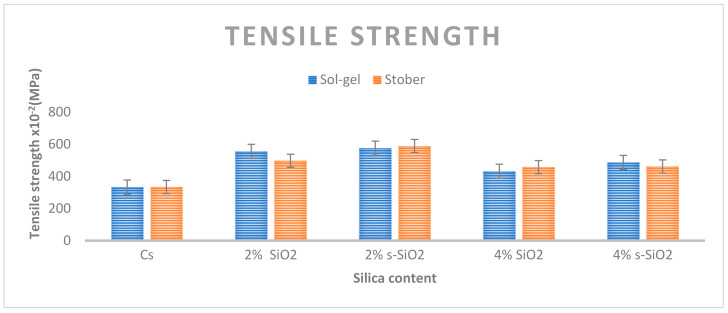
Tensile strength of chitosan membranes.

The mechanical properties of chitosan membranes are shown in Figure 14. The results indicate that the strength of these membranes improved when 2% inorganic filler was added, but it then weakened when 4% was added. The chitosan membranes’ tensile strength was 3.33 MPa before modification. When silica was added, the tensile strength increased to 5.56 and 4.97 MPa. This was followed by a significant increase of 5.76 and 5.89 MPa when 2% s-SiO_2_ Sol-gel and Stober were used, respectively. This suggests that the sulfonation of these particles has a significant impact on the chitosan membranes’ mechanical strength. An increase in the filler content to 4% resulted in a decline in its strength. The lowest value was 4% SiO_2_ (4.32 MPa) Stober. This is consistent with the findings of a 2014 study by Narsito et al., who stated that high silica content can harden a membrane [40]. Silica particles are known to be brittle [40], hence adding more silica to the chitosan matrix breaks its chain entanglement, causing the membrane to have low mechanical strength due to the membrane being hard and stiff, resulting in it fracturing [41,42]. The membrane tensile strength in membranes with silica synthesized through Sol-gel and Stober processes has similar behavior concerning the effect of silica on tensile strength. It can be concluded that the modification of silica with sulfur successfully improves the membrane’s tensile strength. However, it is also important to keep the quantity of silica incorporated into the membrane at a minimum so as to strengthen its mechanical strength.

### 4.9. Oxidation Stability

**Figure 15:**
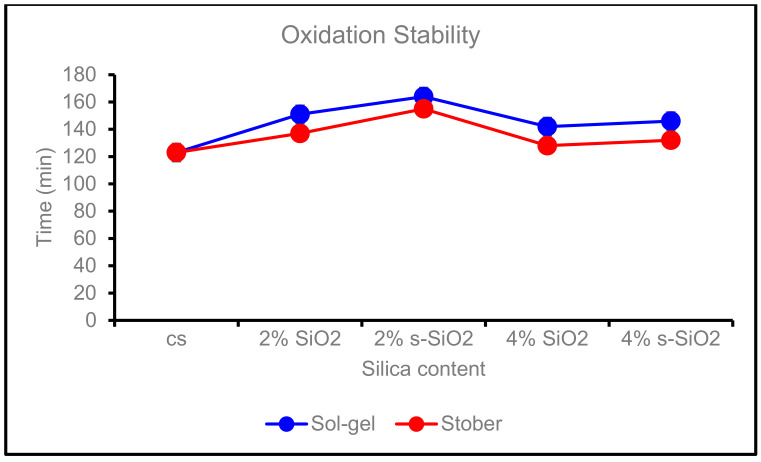
Oxidation stability of Cs, 2% SiO_2_, 2% s-SiO_2,_ 4% SiO_2,_ and 4% s-SiO_2_ at 80 °C.

Figure 15 represents the oxidation stability of chitosan membranes with different types and amounts of silica. The membrane stability test was performed at 80 °C using Fenton reagent 3% H_2_O_2_ containing 3 ppm Fe(SO4)_2_.7H_2_O. The oxidation stability was evaluated as a function of the time the membrane starts to dissociate in the fenton reagent. The unmodified Cs membrane starts to dissociate after 123 min. The incorporation of chitosan with a small amount of silica tends to improve the membrane resistance to chemical attack as the 2% s-SiO_2_ membrane (Sol-gel) degrades after 164 min, followed by the 2% s-SiO_2_ (Stöber process), which degrades after 155 min. However, membranes with high silica content start to degrade at 128 and 142 min on 4% SiO_2_ membranes for Stober and Sol-gel, respectively, making these membranes have the lowest oxidation stability compared with those have a small amount of silica. The decrease in chemical stability of membranes having high silica quantities is due to silica agglomeration in the chitosan matrix reported in Figure 5 and Figure 6. When agglomeration occurs, the membrane’s internal structure becomes weaker in areas where silica aggregates are present, making it more vulnerable to chemical deterioration [43,44]. Temperature also affects the membrane’s rate of deterioration as it increases the system’s energy, which encourages chemical reactions, particularly oxidative activities [45,46]. The composite may degrade more quickly as a result of the accelerated oxidation rate of the chitosan and other components [45,46]. The high temperature might result in the separation of phases or morphological alterations, which can influence how accessible chitosan molecules are to oxidizing agents and impact overall oxidation stability [47].

## 5. Conclusions

The FTIR spectra show no chemical reactions between chitosan and modified silica particles. Instead, a physical interaction between the two occurs. As the elevated silica content increases, its favorable characteristics, such as water uptake and proton conductivity, increase while the methanol permeability decreases. This makes it an ideal material for fuel cells. The amount of water absorbed by chitosan membranes containing silica was observed to increase from 2% to 4% SiO_2_. Silica-based membranes that have been calcined for two hours show an increase in water uptake from 37.9% to 51.97% Stober and from 40% to 56.21% Sol-gel. Sulfonated membranes exhibited exceptional proton conductivities of 0.229 cm/s and 0.234 cm/s on 4% Stober and Sol-gel, respectively. Despite having a lower proton conductivity than membranes modified with sulfonated silica, pure silica-incorporated membranes had a greater proton conductivity than pure chitosan (0.151 S/cm). The reduction in methanol permeability was observed in developed membranes when silica was added, from 2% to 4%. In conclusion, silica-modified chitosan membranes have enormous potential for use in direct methanol fuel cells (DMFC). The addition of silica to chitosan membranes has several benefits that improve their functionality and appropriateness for DMFCs. First, silica enhances the stability and mechanical strength of chitosan membranes. Under the demanding operating circumstances of DMFCs, silica particles support the chitosan matrix, preventing membrane deformation and maintaining structural integrity. The membranes’ durability is increased by this mechanical robustness, which allows them to tolerate mechanical loads. The chitosan membranes with silica particles have better barrier properties against the flow of methanol, which is a critical issue when it comes to DMFCs. By minimizing the crossover between the fuel cell and the membrane, the modified membranes can help improve their efficiency and prevent it from running over the chemical. Furthermore, the addition of silica nanoparticles enhances proton conductivity in chitosan membranes. Silica acts as a proton conductor, facilitating the transport of protons across the membrane and improving the overall cell performance. This increased proton conductivity will contribute to the higher power output and efficiency of the DMFC. Additionally, chitosan membrane proton conductivity was improved by the addition of silica nanoparticles, and proton transport across the membrane was made easier by the role of silica as a proton conductor, which enhances cell function in general. The improvement in proton conductivity and methanol permeability makes the fabricated membranes suitable for applications in fuel cells. It can be concluded that the modification of chitosan with modified silica is necessary to improve the suitability of chitosan in fuel cell technology. Although membrane membranes modified with sulfonated silica show exceptional results in terms of proton conductivity and chemical stability, water uptake is a challenge that still needs to be addressed. However, fuel cell performance must also be improved to support the reliability of the fabricated membranes in fuel cells. Optimizing the synthesis processes and exploring the full potential of chitosan membranes modified with silica for DMFC applications still require additional research and development, which is crucial.

## Data Availability

Not applicable.
